# Lower-extremity inter-joint coordination variability in active individuals with transtibial amputation and healthy males during gait

**DOI:** 10.1038/s41598-024-62655-2

**Published:** 2024-05-22

**Authors:** Alireza Nasri, Ali Abbasi, Zeynab Hadavi, Shahram Abbasi, Zdenek Svoboda

**Affiliations:** 1 Department of Biomechanics and Sports Injuries, Faculty of Physical Education and Sports Sciences, Kharazmi University, Tehran, Iran; 2 Department of Sport Sciences, Faculty of Education and Psychology, Shiraz University, Shiraz, Iran; 3 Faculty of Physical Culture, Department of Natural Sciences in Kinanthropology, Palacky University Olomouc, Olomouc, Czech Republic

**Keywords:** Transtibial amputation, Coordination, Coordination variability, Gait, Biomedical engineering, Motor control

## Abstract

This study was aimed to compare the variability of inter-joint coordination in the lower-extremities during gait between active individuals with transtibial amputation (TTAs) and healthy individuals (HIs). Fifteen active male TTAs (age: 40.6 ± 16.24 years, height: 1.74 ± 0.09 m, and mass: 71.2 ± 8.87 kg) and HIs (age: 37.25 ± 13.11 years, height: 1.75 ± 0.06 m, and mass: 74 ± 8.75 kg) without gait disabilities voluntarily participated in the study. Participants walked along a level walkway covered with Vicon motion capture system, and their lower-extremity kinematics data were recorded during gait. The spatiotemporal gait parameters, lower-extremity joint range of motion (ROM), and their coordination and variability were calculated and averaged to report a single value for each parameter based on biomechanical symmetry assumption in the lower limbs of HIs. Additionally, these parameters were separately calculated and reported for the intact limb (IL) and the prosthesis limb (PL) in TTAs individuals. Finally, a comparison was made between the averaged values in HIs and those in the IL and PL of TTAs subjects. The results showed that the IL had a significantly lower stride length than that of the PL and averaged value in HIs, and the IL had a significantly lower knee ROM and greater stance-phase duration than that of HIs. Moreover, TTAs showed different coordination patterns in pelvis-to-hip, hip-to-knee, and hip-to-ankle couplings in some parts of the gait cycle. It concludes that the active TTAs with PLs walked with more flexion of the knee and hip, which may indicate a progressive walking strategy and the differences in coordination patterns suggest active TTA individuals used different neuromuscular control strategies to adapt to their amputation. Researchers can extend this work by investigating variations in these parameters across diverse patient populations, including different amputation etiologies and prosthetic designs. Moreover, Clinicians can use the findings to tailor rehabilitation programs for TTAs, emphasizing joint flexibility and coordination.

## Introduction

In recent decades, amputation has increased worldwide due to vascular diseases, trauma, and diabetes. According to statistics, the rate of amputation in different regions of the world varies from < 1 in every 10,000 to > 27 per 10,000 Medicare patients, most of which occur in the lower extremities^1^. Individuals with unilateral transtibial amputations have altered gait mechanics and muscle coordination patterns relative to healthy individuals (HIs) because of the necessary prostheses that may cause the onset of joint disorders and asymmetry in gait with prolonged use^2^. Asymmetry in the lower extremities increases the prevalence of secondary disabilities such as osteoarthritis in the intact limb (IL) and prosthesis limb (PL), lower back pain, and falls in individuals with transtibial amputation (TTAs)^3,4^. Koelewijn et al.^5^ demonstrated that gait asymmetries result from compensations required after amputation and the resulting loss of biological ankle function. Significant differences have been reported in the asymmetries between the PL and IL in joint moments^6^, forward propulsion^7^, and ground reaction force^8^ in previous studies on the gait of TTAs. TTAs have a shorter stance phase, longer swing in their PLs than their ILs, and longer stance times in their ILs which may result in greater force impulses in the intact knee joint and increase the risk of arthritis^9^. In addition, altered muscle coordination patterns and increased co-contraction of the quadriceps and hamstring muscles in the PLs of TTAs’ gaits increase knee joint contact forces^10,11^.

The ability to walk with a prosthesis is usually evaluated in symmetry, gait speed, and energy consumption, while gait is a dynamic movement. Consequently, nonlinear dynamic measures such as coordination variability can provide additional insight into the relative timing and magnitude of motion between segments or joints in a kinetic chain^12^. Coordination of human movement is necessary to organize the degrees of freedom of the musculoskeletal system^12^. It is a process in which movement components are gradually organized over time to produce a functional and synergistic movement pattern. However, variability in coordination has been recognized as a critical determinant of the quality of human movement and the flexibility/adaptability of an individual's motor system^13^. HIs have a preferred coordination pattern in their lower extremity joints and segments. They can also access a variety of coordination patterns in response to perturbations or different environmental conditions^12^. However, owing to the lack of limbs and the use of prostheses, TTAs may have a different coordination pattern compared to healthy individuals, and they are less able to respond to gait disturbances^14–18^.

Measures of coordination variability provide information about postural stability, fall risk, injury status, and pathology^12^. Therefore, increasing coordination variability may indicate poor control of the locomotor system, while decreasing it may limit movement^19^. The ability of segment coordination variability to ascertain clinical groups suggests that this variability can be utilized to identify movement patterns that differ from able-bodied individuals. Recent research employing nonlinear dynamic analysis has provided new insights into the biomechanical challenges faced by individuals with amputation. Hu et al.^17^ have contributed to this field by analyzing gait mechanics in individuals with unilateral transfemoral amputations, uncovering compensatory coordination strategies that are instrumental for prosthetic gait rehabilitation. Additionally, their investigation into lower limb coordination during sprinting offers valuable guidance for the development of specialized running prostheses^15^. Complementing these findings, Cheng et al.^14^ demonstrated that a 12-week prosthetic gait training program can significantly enhance walking speed and limb coordination. Esposito et al.^18^ focused on pelvis-trunk coordination across different walking speeds, while Lathouwers et al.^16^ assessed gait patterns using an articulated passive ankle–foot prosthesis, highlighting areas for further research in joint coupling variability during gait in individuals with transfemoral and transtibial amputations. Collectively, these studies underscore the importance of nonlinear dynamic analysis in advancing our understanding of prosthetic gait dynamics. However, these studies used continuous relative phase analysis in individuals with transfemoral amputation and lower limb joint coupling variability in TTAs during gait was not examined by vector coding analysis. Because walking is a dynamic movement, the assessment of joint coordination can be used in the design of prostheses based on joint coupling movement to improve the function of individuals with amputations. Hence, this study was aimed to compare lower extremity joint coordination and its variability during gait between TTAs and healthy individuals. We hypothesized that (1) the values of spatiotemporal gait parameters and joint range of motion (ROM) would be smaller in TTAs than in healthy individuals, (2) the lower extremity joint coordination patterns in the sagittal plane during the gait of TTAs and HIs are different, and (3) the coordination variability in the sagittal plane during gait is different in TTAs and HIs.

## Methods

### Participants

Fifteen active male adults with below-the-knee amputation (age: 40.6 ± 16.24 years, height: 1.74 ± 0.09 m, and mass: 71.2 ± 8.87 kg, type of prosthesis: SACH, years of amputation: 15.53 ± 12.10 years, cause of amputation: trauma) and fifteen able-bodied males without gait disability (age: 37.25 ± 13.11 years, height: 1.75 ± 0.06 m, and mass: 74 ± 8.75 kg) voluntarily participated in this study. The TTAs were recruited from the amputation football team of the Federation of the Disabled of the Islamic Republic of Iran. The primary participant criteria were that subjects were to have used their current prostheses for at least the last six months, experience no pain in the lower extremities, have the ability to walk without using any assistive devices (canes) and have no neuromuscular diseases influencing their standing and walking. In this study, the TTA participants had a K-Level 4 according to the Medicare Functional Classification^20^. HIs were selected from kinesiology students. This study was performed following the Helsinki declaration. All participants signed an informed consent form, and the research protocol and details were approved by the Institutional Review Board of Kharazmi University. All experiments were performed in accordance with relevant guidelines and regulations.

### Gait Data Collection (experimental setup)

A walkway was covered with 10 VICON motion capture system (Oxford Metrics, Oxford, UK) cameras and two force plates (Kistler Group, Winterthur, Switzerland) synchronized with the motion capture system. Three-dimensional kinematic and kinetic data were collected at 120 Hz and 1200 Hz, respectively, while the participants walked along the level walkway. Force platforms were used to identify gait events and collect kinetic data. Twenty reflective markers were attached to the participants’ body landmarks based on the lower-body plug-in gait model. Surface markers were attached directly to the skin or prostheses of the amputees. For TTA subjects, the shank and foot markers on the prosthesis were approximated to match the locations of the corresponding markers on the intact side. The participants stood in an anatomical position to record their static position. We asked participants to walk barefoot at a self-selected speed on an 8-m walkway in the laboratory, and kinematic data were recorded for six gait cycles.

### Data processing

Markers were labeled and gap-filled using Nexus 2.2.3 software (Vicon, Oxford, UK). Afterward, gait parameters were extracted using ProCalc 1.1 (Vicon, Oxford, UK) gait analysis software. Markers trajectories data were low-pass filtered using a fourth-order Butterworth filter with a 6-Hz cut-off frequency. The vertical ground reaction force was used to determine the beginning and end of the stance with a threshold value of 5% body weight^21^. Kinematics and kinetic data were resampled to 100 data points using a custom MATLAB code (MathWorks, Natick, MA, USA).

### Data analysis

The TTA data for PL and IL were calculated separately. The HIs group underwent the same procedure for the left and right limbs, and the average values for the left and right legs were calculated and were compared with the TTA values. Lower extremity joint ROM, spatiotemporal gait parameters, coordination, and coordination variability were calculated and compared between healthy subjects and amputees. The sagittal ROM was calculated for the hip, knee, and ankle by differentiating the maximum from the minimum angle values in each gait cycle, and their mean values were calculated. Inter-joint coordination and its variability (COV) for the pelvis, hip, knee, hip–ankle, and knee–ankle in the sagittal plane were calculated using a modified vector coding technique^22,23^. Coordination patterns were classified into in-phase with proximal dominancy (IPPD), in-phase with distal dominancy (IPDD), anti-phase with proximal dominancy (APPD), and anti-phase with distal dominancy (APDD). In-phase means the two joints move in the same direction, but anti-phase means that two joints move in the opposite direction. The percentage of gait cycle from each coordination pattern were quantified using frequency plots to understand the most prevalent patterns. CV was calculated as the standard deviation of the vector connecting corresponding consecutive time points of the angle-angle plots across all cycles.

### Statistical analysis

We used SPSS software for statistical analysis (IBM SPSS Statistics version 22; SPSS Inc., Chicago, IL, USA). Shapiro–Wilk test were used to screen all data for the normality of distribution. Joint ROMs, spatiotemporal gait parameters, and coordination pattern frequencies over gait cycles were assessed using analysis of variance (ANOVA). Tukey post-hoc tests were used to compare the PLs and ILs with the limbs of HIs. A statistical parametric mapping (SPM) independent t-test was used to detect significant differences in the COV waveforms in all gait cycles between the two groups. The Alpha was set at 0.05 for all statistical analyses. The SPM analyses were implemented using the open-source spm1d code in MATLAB.

## Results

### Joint ROM and spatiotemporal gait parameter results

The IL presented a significantly lower stride length than the PL and HIs. Moreover, the IL had significantly lower knee ROM and greater stance phase duration than that of an HI’s limb (Table1). However, no significant differences between TTAs and HIs were found for hip ROM, cadence, gait speed, stride time, or double support time.

**Table 1:** Spatiotemporal gait data and joint kinematics in sagittal plane.

Parameter	Control (HIs)	Amputees	
		Prosthesis limb	Intact limb
Knee ROM (°)	67.07 (1.23)	58.62 (3.88)	56.34 (3.33)†
Hip ROM (°)	42.08 (1.41)	40.42 (0.83)	38.31 (0.98)
Stride length (m)	1.24 (0.02)	1.22 (0.1)	1.12 (0.2)†**⁕**
Stride time (s)	1.09 (0.01)	1.07 (0.3)	1.07 (0.2)
Gait speed (m/s)	1.13 (0.01)	1.15 (0.3)	1.05 (0.03)
Cadence (step/min)	121.85 (2.35)	119.63 (4.04)	
Stance phase duration (% of cycle)	61.75 (0.48)	62.14 (0.57)	63.71 (0.54)†
Double support time (% of cycle)	12.84 (0.75)	13.20 (0.89)	

^#^Significant difference between prosthesis limb and control group (p ≤ 0.05).

^†^Significant difference between intact limb and control group (p ≤ 0.05).

⁕Significant difference between prosthesis limb and intact limb (p < 0.05).

### Pelvic-to-hip coordination pattern

**Figure 1:**
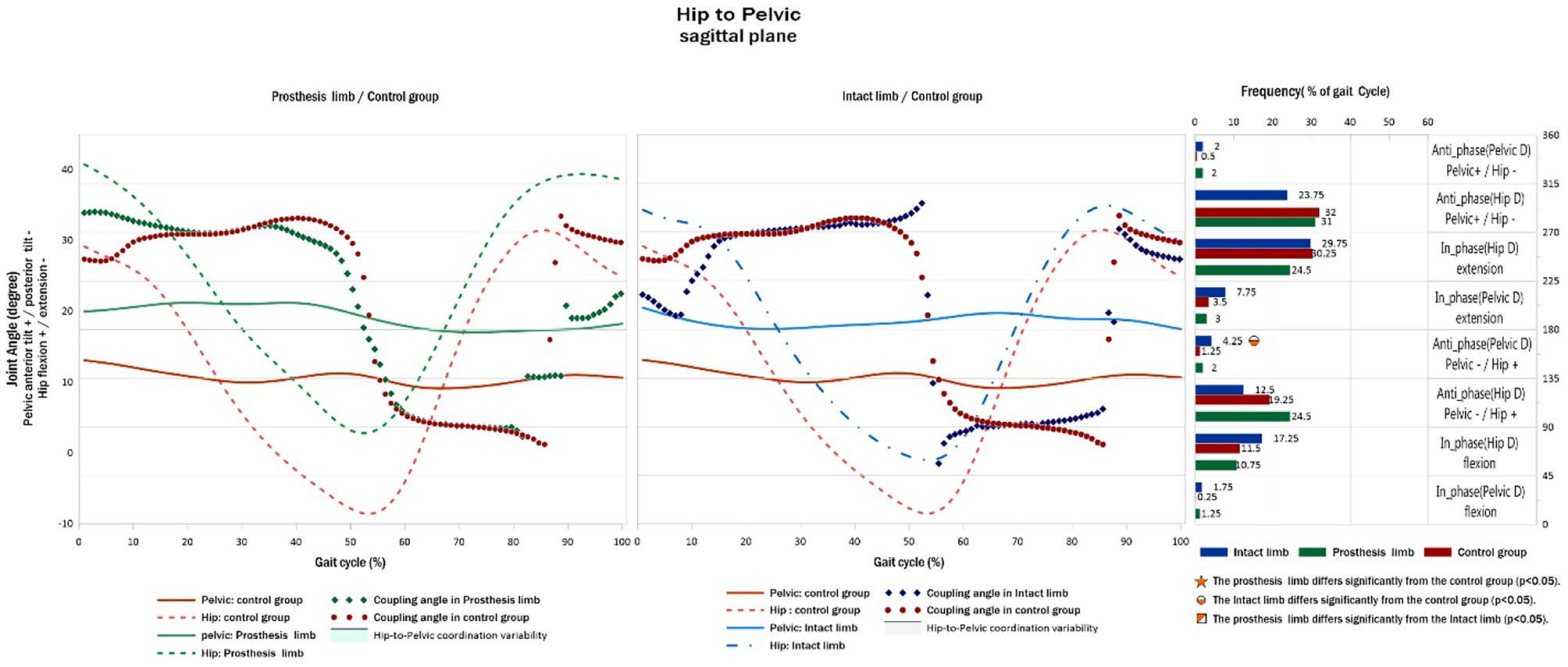
Pelvic-to**-**hip angular displacement diagrams and frequency of coordination patterns (right) in sagittal plane of TTAs and control group.

The frequency of anti-phase with pelvic dominance for the pelvic posterior tilt and hip flexion coordination pattern was significantly lower for the IL of TTAs compared to that of the HI control group limb (Fig. 1).

### Hip-to-knee coordination pattern

**Figure 2:**
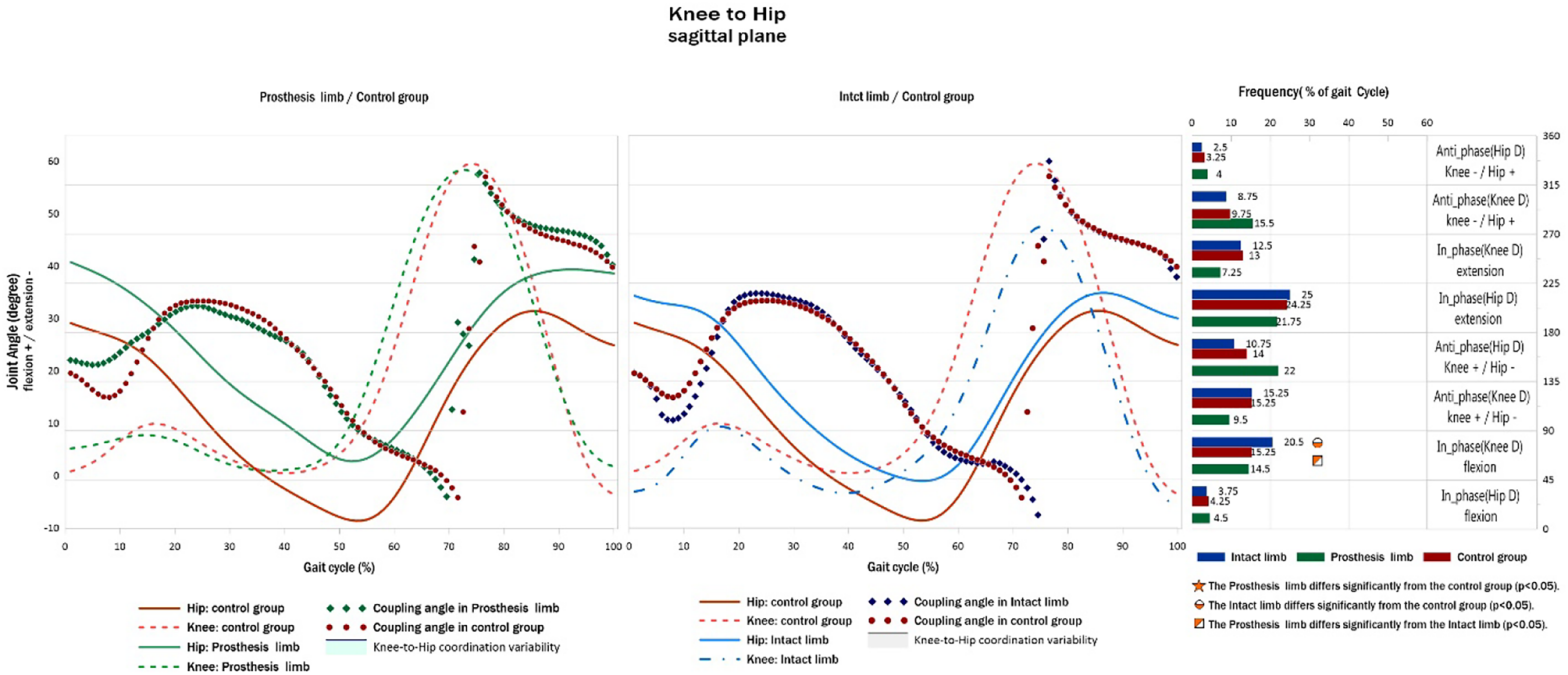
Hip-to-knee angular displacement diagrams and frequency of coordination patterns (right) in sagittal plane of TTAs and control group.

The frequency of in-phase knee dominancy for the knee and hip flexion coordination patterns was significantly lower in the IL group compared to the PL and control groups (Fig. 2).

### Hip-to-ankle coordination pattern

**Figure 3:**
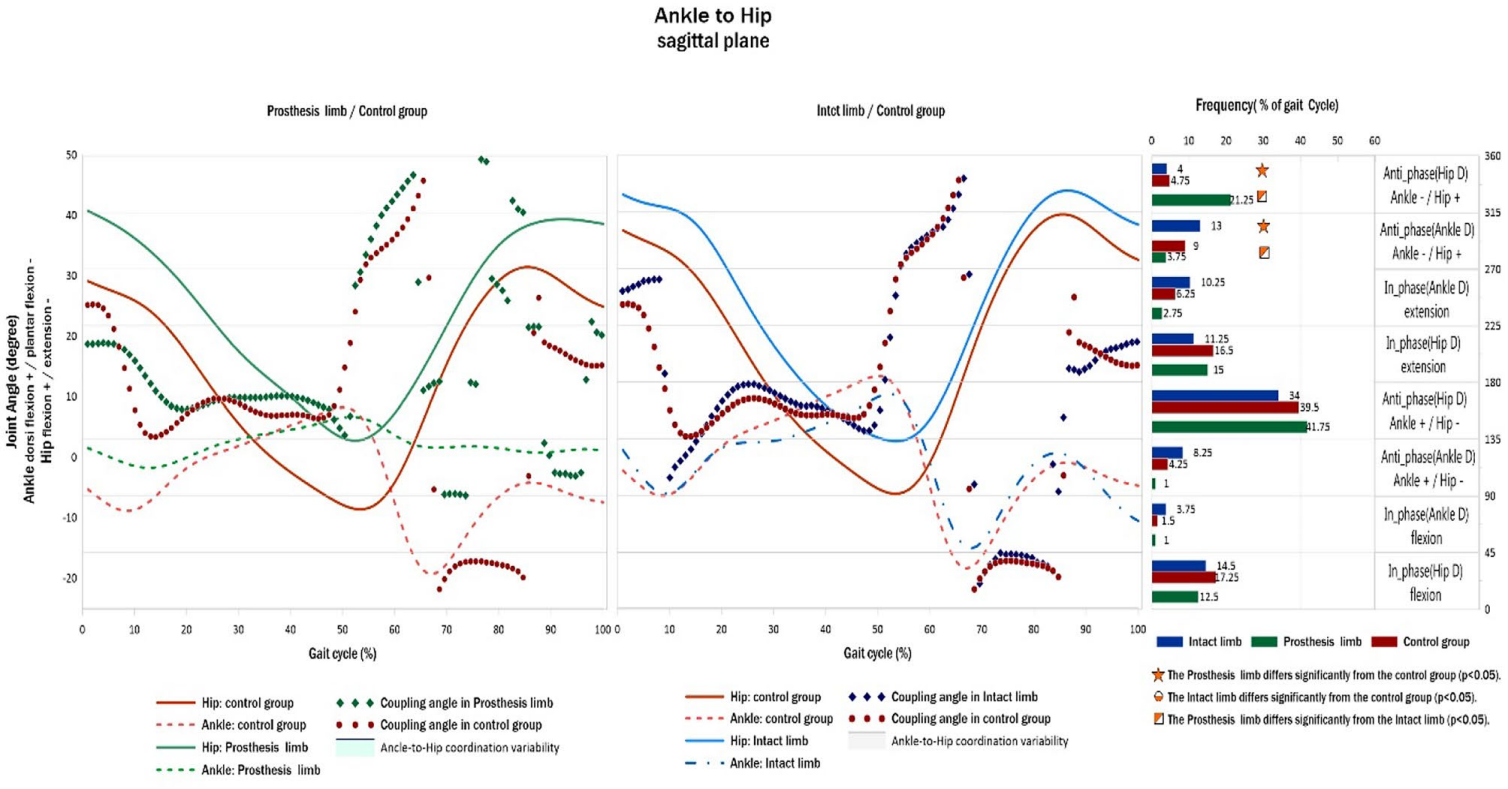
Hip-to-ankle angular displacement diagrams and frequency of coordination patterns (right) in sagittal plane of TTAs and control group.

The frequency of the anti-phase with ankle dominancy for the ankle plantar flexion and hip flexion coordination patterns and that of the anti-phase with hip dominancy for ankle dorsiflexion and hip flexion coordination patterns were significantly lower for the PL in the TTAS group than in the IL and control groups (Fig. 3).

### Knee-to-ankle coordination pattern

**Figure 4:**
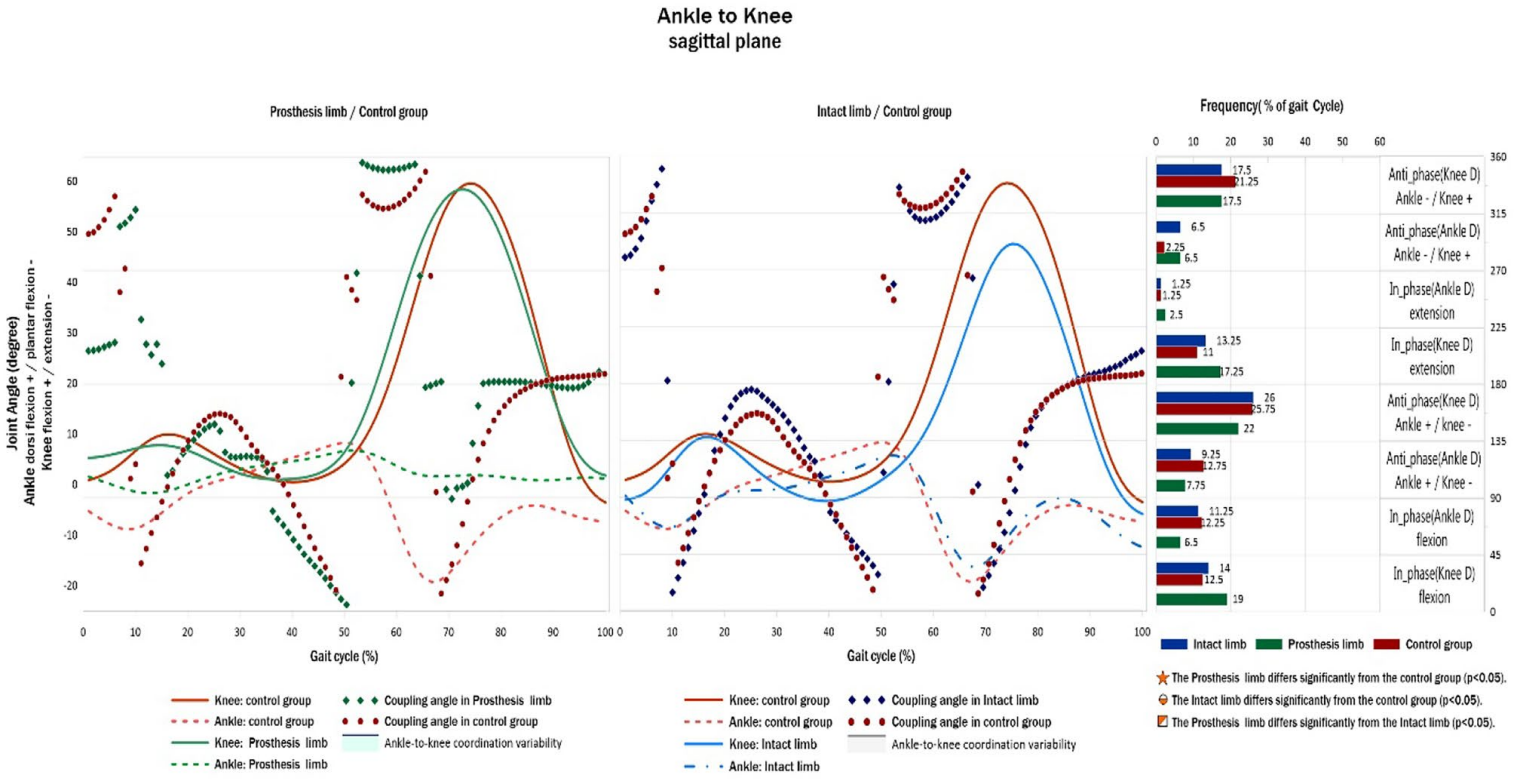
Knee-to-ankle angular displacement diagrams and frequency of coordination patterns (right) in sagittal plane of TTAs and control group.

There was no significant difference in the knee-to-ankle coordination pattern between the TTAs and control groups (Fig. 4).

### Coordination variability results

**Figure 5:**
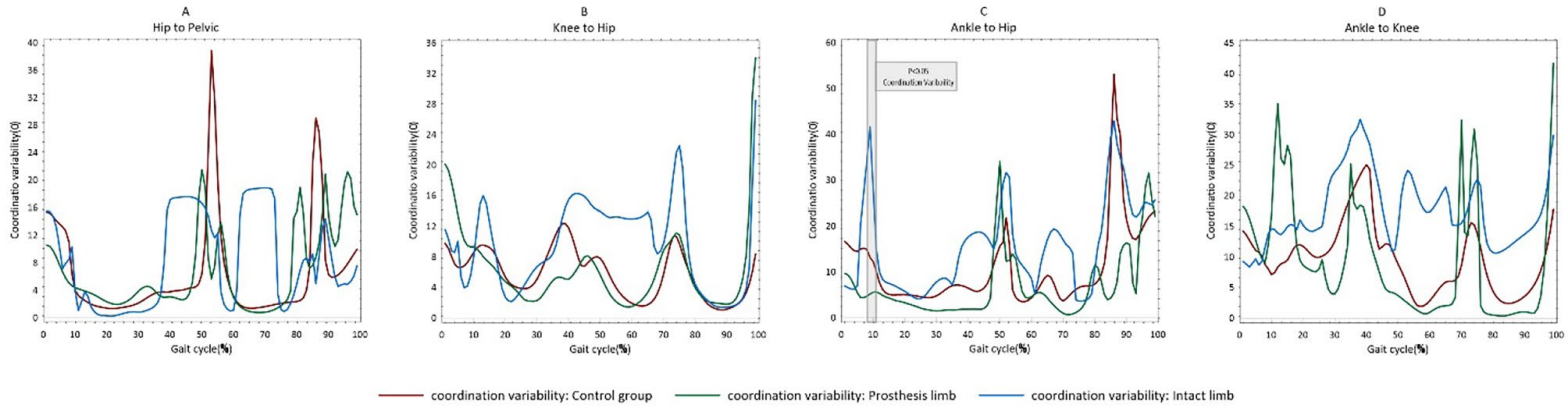
(**A**) Hip-to-pelvic coordination variability, (**B**) knee-to-hip coordination variability, (**C**) ankle-to-hip coordination variability, and (**D**) ankle-to-knee coordination variability during gait cycles in TTAs and control group.

The vector analysis SPM ANOVA results showed that the hip-to-ankle coordination variability for the loading response phase (Fig. 5) was significantly lower for the PL in the TTA group compared with the control group. However, no significant differences between TTAs and HIs were found for hip-to-pelvic, knee-to-hip, or ankle-to-hip coordination variability (Fig. 5).

## Discussion

The main aim of this study was to compare lower extremity joint coordination and its variability in active TTAs and HIs during gait. The active TTA participants in this study exhibited gait patterns that were typically associated with a pathological gait owing to amputation. In addition, the self-selected walking speed is a commonly measured gait parameter often used as an indicator of overall walking performance^24^. The results demonstrated that active TTAs walk with lower ROM in their intact knees than HIs. Moreover, the spatiotemporal parameters significantly differed between the two groups, confirming our first hypothesis regarding the differences in spatiotemporal gait parameters between TTAs and HIs. TTAs had a longer stance phase duration and shorter swing time on their IL compared to HIs. The longer stance and shorter swing phase of the IL were in agreement with previous literature^25^, indicating that less time was spent on the PL because of discomfort or pain^26^.

Moreover, the results showed that active TTAs in the IL walked with a shorter stride length than those in the PL and HIs groups. Our results on stride length were not in line with the study by Bateni^27^, which showed that TTAs’ PL strides were longer than IL strides. The short stride length of the IL may be owing to the compatibility with the exercises that TTAs perform. In this study, no significant difference was observed between the walking speeds of the two groups, but the active TTAs in the IL had a slower walking speed compared with the PL and HIs. Nolan et al.^26^ reported that faster walking speeds in TTAs can increase the asymmetry of loading patterns. Therefore, it appears that TTAs have a slower walking speed to reduce the asymmetry of the loading patterns of the IL. However, our results on walking speed were different from the results of studies by Powers et al.^28^, Schmid-Zalaudek et al.^29^, and Svoboda et al.^21^ that showed significant differences in walking speed between TTAs and HIs. The disparity in our results may be attributed to variations in the physical fitness levels of TTAs. Notably, the TTAs in our research were recruited from an amputation football team, whereas the amputees in the studies by Powers et al.^28^, Schmid-Zalaudek et al.^29^, and Svoboda et al.^21^ were not physically active individuals.

The joint coordination pattern of the lower extremity and its variability in the sagittal plane were significantly different between the active TTAs and HIs in some instances of the gait cycle. This confirms, to some extent, our second and third hypotheses regarding the significant differences in the joint coordination pattern and its variability between TTAs and HIs. The IL showed a more anti-phase coordination pattern in hip flexion-pelvic posterior tilt with pelvic dominancy (135°–180°) compared with the control group. This is related to the greater anterior tilt of the pelvis in the pre-swing phase in the IL than in HIs (Fig. 1). The normal pattern of pelvic obliquity during the weight acceptance phase of an HI’s gait is important for shock absorption^30^. Steven et al.^31^ reported that individuals with bilateral transtibial amputations raised their pelvis during the loading response phase. Consequently, shock absorption during gait may be reduced in individuals with amputation because of reduced knee flexion during the stance phase and an abnormal pelvic pattern. Furthermore, reduced shock absorption in individuals with amputations may contribute to the reported increase in osteoarthritis of the intact knee compared with PL^32^. Active TTAs seem to have an anterior tilt in the pelvis in the IL to reduce shock on the PL during the heel strike and loading response phases.

Our results showed that the IL had a greater in-phase coordination pattern in hip flexion –knee flexion with knee dominancy (45°–90°) compared with the PL and HIs (Fig. 2) that occurred in the initial swing phase. The PL and IL showed more hip flexion compared with HIs during all gait cycles, particularly in the late stance and initial and late swing phases. Furthermore, the PL showed more knee flexion than the control group at late stance, but IL showed more extension at all gait cycles, particularly at late stance (Fig. 2). In the gait of HIs, the plantar flexors and knee extensors are the primary sources of propulsion^33^. Moreover, hip flexor activity during push-off is a major contributor to limb propulsion, as Sadeghi et al.^34^ reported increased power generation in the hip flexors during 50–60% of the PL gait cycle. In TTAs without plantar flexors on one limb, knee extensors may become more important for producing forward propulsion and can help elevate the centre of mass and reduce the probability of foot clearance in the PL. Moreover, hip flexors have become more important for producing hip flexion to initiate the swing phase and reduce the likelihood of foot clearance during the swing phase. Thus, the lack of ankle plantar flexors may compensate for the increased hip and knee flexion of a PL and increase knee extension of IL.

The results also showed that the PL had a less anti-phase coordination pattern in hip flexion–ankle plantar flexion with ankle dominancy (270–315) compared with IL and HIs (Fig. 3), which occurred in the terminal stance and pre-swing phases. This pattern indicates that PL have less hip flexion and ankle plantar flexion during the terminal stance and pre-swing phases owing to the use of solid prostheses and the lack of ankle plantar flexion. Moreover, the PL exhibited a more anti-phase coordination pattern in hip flexion–ankle plantar flexion with hip dominancy (315°–360°) compared with the IL and HIs and occurred in the pre-swing and initial swing phases (Fig. 3). Efficient gait and mobility are often restricted by the discomfort and functional limitations of a prosthesis, and active TTAs typically employ compensatory mechanisms owing to a lack of power generation in the ankle joint^35^, while the ankle moment plays an essential role in gait propulsion^36^. This pattern indicates that the PL has a greater hip flexion during the pre-swing and initial swing phases. This coordination pattern occurs due to the lack of plantar flexion in the PL during pre-swing and must be compensated for with more hip flexion. Furthermore, increased hip flexion during the initial swing helps reduce the probability of foot clearance.

Although TTAs could not achieve plantar flexion at their PL, there were no significant differences in knee-to-ankle coordination patterns in all gait cycles between healthy subjects and active TTAs (Fig. 4), perhaps because there were only fifteen TTA participants in the study. Coordination calculations using a larger number of TTAs may yield different results. Moreover, the results showed that PL had less coordination variability in all coupling patterns (Fig. 5). Previous studies reported lower coordination variability in the lower extremity joints in the sagittal plane during the stance phase of gait in fallers than in non-fallers^37^. Then less coordination variability in joint coupling indicates a lower degree of freedom and less capability to adapt to perturbations during gait and increases the fall risk of TTAs. The significantly greater coordination variability in IL at initial contact may indicate a greater degree of freedom for IL during initial contact to adapt to the restricted ankle motion in the PL during late stance and may increase TTAs’ ability to load responses on their IL.

Our conclusions must be interpreted with the awareness of the following limitations. First, active TTA participants were not matched according to the cause of amputation or age. Therefore, generalization of these findings to a broader population is difficult. In addition, we examined the joint coordination pattern and its variability only in the sagittal plane. However, investigating coordination patterns in joints and segments in the frontal and horizontal movement planes may provide a better insight regarding the mechanics of lower extremity joint coupling patterns and their variability in TTAs.

## Conclusion

This study showed that active TTAs in the IL have slower walking speeds with more stance phase and double support time than HIs and may result in greater force impulses in the intact knee joint and increase their risk of arthritis. Moreover, TTAs in the PL walked with more flexion of the knee and hip and may indicate a progressive walking strategy. Active TTAs showed different coordination patterns in the pelvis-to-hip, hip-to-knee, and hip-to-ankle couplings in some parts of the gait cycle. This suggests that active TTAs use a different neuromuscular control strategy to adapt to their amputations. Researchers can extend this work by investigating variations in these parameters across diverse patient populations, including different amputation etiologies and prosthetic designs, or they can delve deeper into these strategies, potentially informing rehabilitation protocols and prosthetic training. Moreover, clinicians can use the findings to tailor rehabilitation programs for TTAs, emphasizing joint flexibility and coordination.

## Data Availability

The datasets used and/or analysed during the current study available from the corresponding author on reasonable request.
